# Supplementary material for: Learning curve and surgical outcome of robotic assisted colorectal surgery with ERAS program
Source: Sci Rep. 2022 Nov 29;12:20566. doi: 10.1038/s41598-022-24665-w (PMC9709162; doi:10.1038/s41598-022-24665-w)
Supplement: Supplementary file 1 — Supplementary Information 1. [file 41598_2022_24665_MOESM1_ESM.zip › LSC Raw data-Ñ[▒K/094/O_ñw╝╨░O▒Kñσ.pdf]

正式摘要(電子簽章：0000001996520l1)

請選擇...reporter

臺中榮民總醫院

Operation Note

[redacted]

- 手術日期：2020/09/24
- 手術醫師：VS 蔣鋒帆，CR 陳映菁，Fellow 林昌霖，R 楊捷儒
- 執行麻醉：VS 徐煥
- Anesthesia：GE
- Position：Supine
- Aseptic agents：Soap，Betadine Alcohol，Betadine Aqua

Diagnosis

- Pre-operative diagnosis : T COLON CA SP LOOP T COLOSTOMY, GBS
- Post-operative diagnosis : Ditto.

Surgical Indication

- For malignancy removal and pathologic proof

Operation

- 1. Laparoscopic right hemicolectomy with side to side anastomosis, endo GIA 45-3.5mm*3, re-inforced with 3-0 silk lamber suture.
  2. Segmental resection of small intestine, with side-to-side anastomosis with endo GIA 45.3.5mm*2, re-inforced with 3-0 silk lamber suture.
  3. Closure of T loop colostomy
  4. Vessel ligation: ileo-colic and right branches of middle colic vessels.
  5. Cut margin: proximal 19cm(colon), 5cm(ileum), distal 5cm.
  6. Laparoscopic cholecystectomy

Finding

- 1. Severe adhesion of T-colostomy with wound and fascia.
  2. A 3*4cm Borrmann type II annular ulcerative tumor at middle transverse colon, which invaded to the muscular layer, but didn't penetrate through the serosa. There were some enlarged lymph nodes at mesocolon, cT3N1M0.
  3. There was no visible liver metastasis
  4. peritoneal seeding(+)
  5. One mass (lymph node? peritoneal seeding) invade mesocolon of cecum and samll intestine
  6. No synchronous polyp or tumor at resected specimen.
  7. pigmented GBS

Procedure

- General endotracheal anesthesia was induced and then the patient was placed in the supine position. Preoperative antibiotics were given. A Foley catheter and nasogastric tube were placed. The abdomen was prepped and draped in standard sterile fashion. Colostomy was taken down from abdominal wall, and then was sutured by 1-0 vicryl to avoid intestinal content contamination. Wound retractor was applied through this wound. 12mm balloon trocar was inserted into the peritoneal cavity, pneumoperitoneum was established with CO2 inflation. Laparoscope was inserted, four 5mm trocars were inserted over RLQ *2 and LUQ, LLQ of abdomen. The liver, omentum and peritoneum were inspected for evidence of metastatic disease. No metastatic disease was noted. The small bowel was inspected and retracted to downward by grasper. The peritoneum overlying the mesentery was then scored with electrocautery and the ileocolic artery and right branch of middle colic vessels were identified, ligated with endoclips, and transected. The remaining vessels to the

- cecum were also divided and ligated. Using electrocautery, omentum was freed from the transverse colon and lesser sac was entered. The colon was freed from its peritoneal attachments along the line of Toldt from the cecum to the hepatic flexure. Hemostasis was checked in the operative field. Cecum, ascending colon and hepatic flexure were mobilized and then laparoscope was removed. The ascending colon was delivered into the previous colostomy incision. A segment of small intestine was adhesioned with cecum tightly, and unable to separate it, mesentary of small intestine was ligated, 5cm segmental small intestine resection and re-anastomosis with endo GIA 45-3.5mm*2. The points of transection were selected proximally at termial ileum and distally(5cm distal to the tumor). The bowel was divided. The two ends of the bowel were checked and found to be viable, with excellent blood supply. The fat was gently cleared from the terminal 2-3 mm of the bowel ends. The two ends of the bowel were brought into apposition and found to lie comfortably without excessive tension. Endo GIA 45-3.5*3 were applied to make side-to-side ileocolic anastmosis, an outer layer of interrupted 3-0 silk Lambert sutures was done for re-inforcement. The anastomosis was checked and found to be intact and widely patent. Then we consulted GS doctor for laparoscopic cholecytectomy. Subxyphoid incision was made and 12mm trochar was inserted, after cholecystectomy. GB was taken out from previous colostomy wound. The fascia of previous colostomy wound was closed with interrupted sutures of No.1 Vicryl, and wound was closed with 2-0 vicryl. The fascia of the 12 mm trocar wound was closed with 1-0 Dexon. Subcutaneous tissues were closed with interrupted 3-0 Nylon. The patient tolerated the procedure well and was brought to the POR unit in stable condition.
- Estimated blood loss: 100 c.c
- 器械數量: 正確
- 紗布數量: 正確

Image

- PAINT
  - colon & rectum

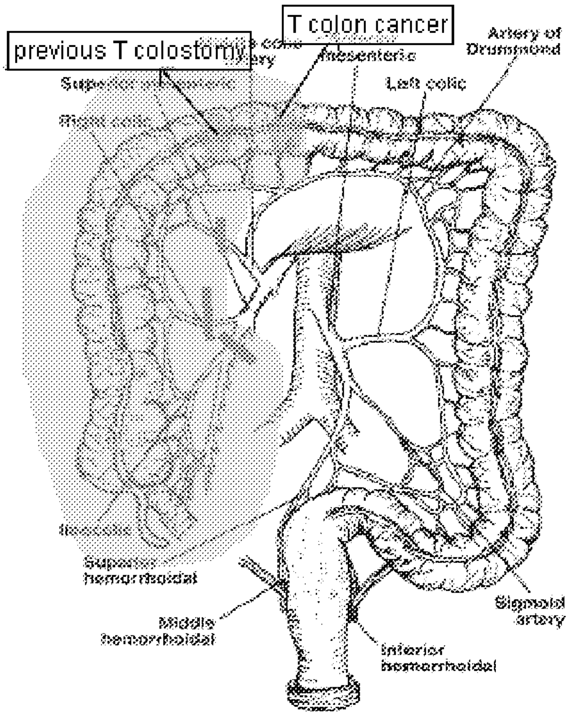

  - abdomen

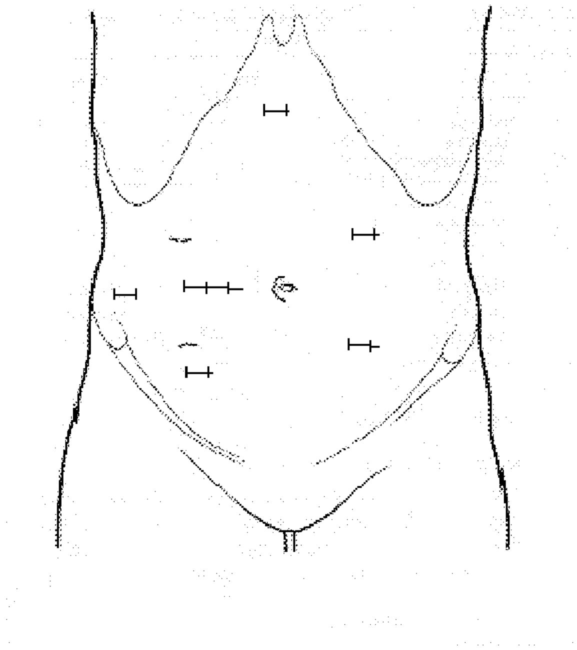

主任 2062F蔣鋒帆 / 醫師 2303K陳映菁

正式摘要(電子簽章：000000199652011)

請選擇...reporter

doctorRank:B
